# The activity of the Research Investments in Global Health study and ways forward within the global funding and policy landscape

**DOI:** 10.1186/s12919-016-0065-2

**Published:** 2016-10-10

**Authors:** Michael G. Head, Rebecca J. Brown

**Affiliations:** 1 Global Health Research Institute and Faculty of Medicine, University of Southampton, Southampton, UK; 2 Farr Institute for Health Informatics, University College London, London, UK

## Abstract

The Research Investments in Global Health (ResIn, www.researchinvestments.org) study analyses funding trends in health research, with a predominant focus on infectious diseases. Since October 2015, the project is funded by the Bill & Melinda Gates Foundation and is now based at the University of Southampton in the UK. In 2016, Public Policy@Southampton provided ResIn with a small grant to explore developing links with policy, funding and research stakeholders with an interest in global health. Three meetings were organised in London (Wellcome Trust, 25 May 2016), Brussels (UK Research Office, 2 June 2016), and Geneva (WHO R&D Observatory, 8 June 2016). In total, 45 stakeholders attended and provided comment and critique on the study methodology and potential expansion into other disciplines. A theme that emerged across all three meetings concerned the use of a standardised categorisation system. A key benefit of the ResIn study is the ability to present granular detail in precise areas. Further work packages that could enhance the use of the collected R&D data included integration with geospatial, policy and scientometric methodologies. There was broad enthusiasm that outputs from these proposed projects would provide clear benefits in informing health policy and R&D strategy. Outputs from the ongoing study covering infection-related R&D investments in the G20 nations will be available in 2017.

## Background

The Research Investments in Global Health study (ResIn, www.researchinvestments.org) has systematically mapped the UK landscape for infectious disease research by describing funding trends for awards to UK institutions between 1997 and 2013 inclusive [1, 2]. Awards related to infection from all the major funders of health and biomedical research were categorised by disease area, pathogen, clinical specialty and the type of science along the R&D pipeline. Investments were also compared to the global burden of disease, using data sourced from the Global Burden of Disease (GBD) study [3, 4], to gain an idea of UK areas of likely research strength and diseases that appear relatively poorly-funded.

Since October 2015, ResIn has been funded by the Bill & Melinda Gates Foundation to consider R&D investments for infection across all the G20 nations. In 2016, Public Policy @ Southampton (http://www.southampton.ac.uk/publicpolicy) awarded ResIn a small grant (£3900) to organise three workshops that engage funders, policymakers and other stakeholders in global health. With a combined attendance of 45 individuals (Tables 1, 2, and 3), the aim of the workshops was to highlight the activity of the ResIn project, to prepare the R&D community for the publication of the global dataset in 2017, to present ideas for expansion into other disciplines, and to seek critique and feedback on study methodology.

**Table 1:** Invited attendees of London workshop, 25 May 2016

Alex	Blum	Public Policy @ Southampton
Gavin	Costigan	Public Policy @ Southampton
Catherine	Cotton	Federation of European Microbiological Societies
Kevin	Dolby	Wellcome Trust
Emily	Gale	Medical Research Council
Pat	Goodwin	Microbiology Society
Felix	Greaves	Public Health England
Chris	Lowry	British Society of Immunology
Anthony	Scott	London School of Hygiene & Tropical Medicine
Andrew	Smith	Foreign and Commonwealth Office
Vinny	Smith	Meningitis Research Foundation
Neil	Squires	Faculty of Public Health
Sophie	Taysom	Department of Health
Charlotte	Watts	UK Department for International Development
Philip	Price	Wellcome Trust
Graham	Tynan	Wellcome Trust
Marco	De Ambrogi	The Lancet Infectious Diseases
John	Broughall	Antibiotic Research UK
Mark	Zuckerman	Clinical Virology Network

**Table 2:** Invited attendees of Brussels workshop, 2 June 2016

Jozef	Anne	Federation of European Microbiological Societies
Brendan	Barnes	European Federation of Pharmaceutical Industries and Associations
Roberto	Bertollini	WHO Brussels office
Julie	Cantalou	Public Policy@Southampton
Laurence	Colin	European Research Council
Alain	Deleener	Research Foundation - Flanders (FWO)
Evelyn	Depoortere	European Commission
Maribel	Glogowoski	UK Research Office
Oliver	Karsten	Friends of the Global Fund Europe
Barbara	Kerstiens	European Commission
Hugh	Laverty	Innovative Medicines Initiative
Kevin	McCarthy	European Commission
Martine	Sabbe	Scientific Institute of Public Health

**Table 3:** Invited attendees of Geneva workshop, 8 June 2016

Taghreed	Adam	WHO Global Observatory on Health R&D
Lauranne	Botti	COHRED
Vania	de la Fuente Nunez	WHO Global Observatory on Health R&D
Nebiat	Gebreselassie	WHO Global TB Programme
Abdul	Ghaffar	WHO Alliance for Health Policy and Systems Research
Hope	Johnson	GAVI
Christian	Leindhart	WHO Global TB Programme
Manuel	Martin	UAEM
Maya	Matthews	European Commission
Deepak	Mattur	UNAIDS
Amit	Prasad	WHO Global Observatory on Health R&D
Alistair	Robb	WHO Information, Evidence and Research
Robert	Terry	Special Programme for Research and Training in Tropical Diseases (TDR)

Discussions centred around two themes – methodology, and wider engagement with the research, funding and policy communities. The material presented at the workshops is provided here (supplementary information).

## Methodologies

A theme that emerged across all three meetings concerned the use of a standardised categorisation system– comment included that ResIn should adopt an existing system (such as that used by the NIH, Health Research Classification System or MESH terms) and work towards a unified categorisation methodology. The ResIn project created its own system which would cover many of the keyword and disease areas used by the other classifications; however, there was general agreement that it would be useful if a standardised system could be introduced that remained comprehensive but with flexibility to adapt as research investment analyses evolve. This could be an activity developed in collaboration with the WHO R&D Observatory.

Further category development would be useful to draw out further granular detail for highlighting specific sub-sections of data, for example further breakdown of the public health category to illustrate investments directed towards social science, epidemiology, economics etc; one further example was to differentiate between primary data collection and secondary data analysis. A possible weighting system to allocate proportional amounts of funding across pathogens was suggested e.g. £1 m study relating to co-infection of HIV and tuberculosis would see £500 k towards HIV and £500 k towards tuberculosis. This would be more difficult to achieve across cross-cutting themes (such as global health and antimicrobial resistance). The distribution of funds from lead institutions to collaborators (particularly those in low- and middle-income countries (LMICs)) is currently difficult to systematically track but is an important factor to consider. Additional useful analyses would include comparison of levels of investment with i) risk factors for disease (alongside the existing burden of disease comparisons), ii) implementation and aid funding; iii) projected future health burdens; iv) differences between datasets of disease burden e.g. that produced by the Institute for Health Metrics and Evaluation and the WHO; v) awards directed straight to LMICs, including infrastructure and other capacity-building initiatives.

The data gap of private sector data was noted, with limited options for systematic provision of such information. Pragmatic approaches may help (for example methods developed by Prof Jonathan Grant, King’s College London). Partnerships between the private sector and public or charitable organisations (such as the International Medicines Initiative or GAVI) tend to provide some public information about industry investment. Policy Cures have also carried out surveys with the for-profit sector to obtain such information anonymously.

Differing levels of overheads across different sectors will mean that the amount of money for the visible costs of research (staff, consumables) varies between funders. A sensitivity analysis to assess these differences would be helpful. Another area of difficulty highlighted was the funding flow between lead institution receiving the award and project partners – at any large systematic scale, this would be very complex to describe. Accessibility of data across countries is anticipated to be broadly satisfactory, though there may be language difficulties with some nations (Russia and China were noted examples where open data, in English, may be limited). Investments directed specifically towards infrastructure to house research activity is not directly captured as part of the ResIn analysis, and is often difficult to attribute to disease areas.

## Project engagement with global and national stakeholders

The ResIn analysis can drive forward the movement towards open access investment data, in an accessible format. Funders and other stakeholders could be invited to suggest further aspects of categorisation and data visualisations that would be most useful. The granularity of the data is important, and the ability to provide detailed cross- and sub-national analyses is extremely useful. The format in which ResIn data was made available was discussed, with reports and papers, policy briefs and online customisable visualisations all considered useful, depending on the audience. The online visualisations, in particular, allow users to draw data and infer conclusions in precise areas.

The presentation included possibilities for further work packages that could enhance the data collection, including integration with geospatial, policy and scientometric methodologies. There was broad enthusiasm that outputs from these proposed projects would provide clear benefits in informing health policy and R&D strategy. Expert input from colleagues and organisations in resource-poor settings could identify priority projects and areas of focus to be addressed by the ResIn study.

There was a mixed view on whether expansion of the ResIn project should concentrate on a broad focus of large-scale data collection and top-level analyses, or a more defined focus on specific topic areas (e.g. infectious disease) where the project can provide significant expertise. A networking function that proactively links stakeholders across national borders and disease areas was also suggested as a potential benefit.

The presentation noted that funding was very reactive in nature, and there followed suggestions that ResIn could help to inform proactive thinking about future R&D priorities and work alongside existing groups that have a ‘horizon-scanning’ remit (such as the EU network for emergency preparedness). Broader engagement with the WHO would be particularly important, and engagement with some member nations will typically be most effective if in conjunction with the WHO.
